# Induction of Resistance Mediated by an Attenuated Strain of *Valsa mali* var. *mali* Using Pathogen-Apple Callus Interaction System

**DOI:** 10.1155/2014/201382

**Published:** 2014-06-25

**Authors:** Qingming Zhang, Caixia Wang, Daojing Yong, Guifang Li, Xiangli Dong, Baohua Li

**Affiliations:** ^1^College of Chemistry and Pharmaceutical Sciences, Qingdao Agricultural University, Qingdao 266109, China; ^2^College of Agronomy and Plant Protection, Key Lab of Integrated Crop Pest Management of Shandong Province, Qingdao Agricultural University, No. 700 Changcheng Road, Qingdao 266109, China

## Abstract

To study the induced resistance in apple against *Valsa mali* var. *mali (Vmm)*, a *Vmm*–apple callus interaction system was developed to evaluate the induced resistance of an attenuated *Vmm* strain LXS081501 against further infection by a virulent *Vmm* strain LXS080601. The infection index was up to 97.32 for apple calli inoculated with LXS080601 alone at 15 days after inoculation whereas it was only 41.84 for calli pretreated with LXS081501 followed by LXS080601 inoculation. In addition, the maximum levels of free proline, soluble sugar, and protein in calli treated with LXS081501 plus LXS080601 were 2.14 to 3.47 times higher than controls and 1.42 to 1.75 times higher than LXS080601 treatment. The activities of defense-related enzymes such as phenylalanine ammonia lyase (PAL), polyphenol oxidase (PPO), peroxidase (POD), and catalase (CAT) as well as *β*-1,3-glucanase and chitinase in apple calli inoculated with LXS080601 alone or LXS081501 plus LXS080601 increased significantly 24 hai and peaked from 48 to 120 hpi. However, in the latter treatment, the maximum enzyme activities were much higher and the activities always maintained much higher levels than control during the experimental period. These results suggested the roles of osmotic adjustment substances and defense-related enzymes in induced resistance.

## 1. Introduction

Apple tree canker disease, caused by* Valsa mali *var.* mali* (*Vmm*), is a destructive disease that is prevalent in main apple orchards in China. Cankers usually occur on branches and tree trunk, eventually leading to the death of the whole tree and sometimes the failure of the whole orchard [1]. Official investigation revealed that, in 2008, the incidence of apple canker disease was 52.7% across China [2]. In addition, apple orchards in Shandong province suffered from severe disease in 2011 and a fifth epidemic was predicted to threaten the apple production in China [3, 4]. As a weak pathogen,* Vmm* invades apple tree by secreting cell wall degrading enzymes and toxins to collapse and kill host cells [5–8]. Presently, there are no apple cultivars known to be resistant to* Vmm *at satisfactory levels [9, 10]. To make the consequence of this disease worse, the pathogen also invades xylem and resides there without inducing symptoms, rendering the use of fungicide ineffective [4].

As an alternative to traditional control method, it is of great interest to employ induced resistance in plants to combat economically important plant diseases [11, 12]. It is well documented that some plants previously inoculated with attenuated or avirulent pathogens become resistant to further infection, suggesting that plants can mount an efficient and active defense [13]. To this end, the understanding of the mechanisms by which plant resistance is activated via pathogens is critical for the development of efficient biocontrol [14, 15]. Salazar et al. [14] reported an avirulent strain F7 of* Colletotrichum fragariae* that was able to induce defense responses against anthracnose caused by a virulent isolate of* C. acutatum* in strawberry plants. In this case, protection effects were accompanied by a rapid accumulation of the reactive oxygen species including hydrogen peroxide and superoxide anion in leaves [14]. Similarly, Shishido et al. [16] utilized the strain Fo-B2 of* Fusarium oxysporum* as a biological agent to control* Fusarium* wilt disease on tomato.

Apple canker disease usually displays symptoms on branches and trunk. However, there are several shortcomings when utilizing branches as experimental materials for research, due to variations in branch ages and water content as well as the sampling times. To circumvent these disadvantages, we established a stable* Vmm*-apple callus interaction system for disease evaluation in laboratory. In this system, the induction of resistance in apple calli mediated by the attenuated* Vmm* strain LXS081501 against a virulent* Vmm *strain LXS080601 was verified. To gain an understanding of the underlying mechanisms, osmotic adjustment substance contents and elicitation of defense-related enzymes were evaluated in apple callus challenged with attenuated strain LXS081501, virulent strain LXS080601, or both. This would be the first report to describe induced resistance in apple mediated by attenuated pathogenic fungi.

## 2. Materials and Methods

### 2.1. Plant Material and Fungal Strains

Two apple cultivars, “Fuji” and “Gala” (*Malus domestica* Borkh.), were used in the experiments. “Fuji” and “Gala” are main varieties grown in China but are susceptible to* Vmm*. Five-year-old apple trees were grown in a greenhouse under natural daylight conditions.

A virulent strain LXS080601 was obtained from infected “Fuji” trees and an attenuated strain LXS081501 from diseased Chinese flowering crabapple (*M. spectabilis*). Strains were single-hyphen propagated to obtain pure cultures on PDA medium (potato dextrose agar) and maintained on PDA slants at 4°C [8].

### 2.2. *In Vitro* Plantlet Culture and Callus Induction

“Fuji” stems with axillary buds or “Gala” stems with dormant buds were selected as explants to establish* in vitro* regeneration systems [17]. Specifically, “Fuji” and “Gala” stems were cleaned with tap water and cut into pieces with one or two buds on each piece. Sterilization was performed by using 0.5% sodium hypochlorite for three to five minutes under laminar hood, after which these stem pieces were washed with sterile water for several times followed by treatment with 75% ethanol for three minutes and four-time washes with sterile water to remove ethanol residues. After sterilization, “Fuji” stems with axillary bud and “Gala” stems with dormant bud scales removed were inoculated into induction medium for plant regeneration and the medium recipe was basic Murashige and Skoog (MS) medium [18] supplemented with 2.0 mg/L 6-benzyladenine (BA), 0.2 mg/L 3-indolebutyric acid (IBA), 1.0 g/L vitamin C (VC), 4.0 g/L polyvinylpyrrolidone (PVP), 30 g/L sucrose, and 6.0 g/L agar, pH 5.8. Regenerated plantlets were propagated and maintained on subculturing medium (MS medium plus 1.0 mg/L BA, 0.1 mg/L IBA, 1.0 g/L VC, 4.0 g/L PVP, 30 g/L sucrose, and 6.0 g/L agar, pH 5.8). The plantlets were subcultured every 4 weeks. The culturing conditions were 25 ± 1°C, 75% relative humidity, and 16 h photoperiod with the light intensity as 20 *μ*mol/m^2^/s.

Leaves from the* in vitro *regenerated plantlets were used for callus induction according to the method of James et al. [19] with modifications. The excised leaves were sliced vertically to the main vein and placed on callus-inducing medium with the abaxial side facing down. The callus-inducing medium was basic MS medium with 1.0 mg/L thidiazuron (TDZ), 0.2 mg/L IBA, 0.8 mg/L 2, 4-D, 1.0 g/L VC, 4.0 g/L PVP, 30 g/L sucrose, and 6.0 g/L agar (pH 5.8). Callus was induced under darkness and the appearance of yellowish tissues around wounds indicated successful induction of callus, which was transferred to subculturing medium for maintenance under the same conditions with plantlet culturing. The calli were subcultured at three-week interval. The recipe of subculturing medium for callus was similar to that of callus-inducing medium but with half levels of hormones.

### 2.3. Induction of Resistance by an Attenuated* Vmm* Strain

*Vmm* strains LXS080601 and LXS081501 were recovered on PDA plates for 3 days at 25°C under darkness, from the margins of which plugs with a diameter of 4 mm were made with a cork borer and used as inocula [20]. Under sterile conditions, calli with similar growing status and sizes (~12 × 15 mm) were placed into 2% water agar plate with a diameter of 90 mm. Plugs of LXS081501 were inoculated into one side of calli and, 48 hours later, the plugs of LXS080601 were placed into the other side of calli and, at the same time, plugs of LXS081501 were discarded. Calli inoculated with either LXS080601 or LXS081501 alone served as control. Sealed Petri dishes were incubated at 25°C with a 12 h photoperiod, and symptoms were carefully examined. Each treatment was done in triplicate, with ninety calli randomly selected and placed into water agar plates (five in each). This experiment was performed twice.

After inoculation, disease symptoms were recorded and classified according to disease severity rating (DSR) standards established by Liang et al. [21] with some modifications. In our cases, DSR standards were assessed using the following scale: 0 = callus remained unaffected with no hyphae growth; 1 = hyphae infected less than half area of callus with slight browning; 2 = the diseased area was between half and two-thirds of the callus with partial water-soaking; 3 = whole callus was covered with thin hyphae and displayed browning; 4 = massive hyphae appeared on the whole callus that became severely water-soaked and dark-brown. The infection index was evaluated at 3, 6, 9, 12, and 15 days after pathogen inoculation. Infection index is given by

(1) $$\begin{array}{c} \frac{\sum \left(\text{DSR}\;\text{scale}\times \text{number}\;\text{of}\;\text{calli}\;\text{within}\;\text{each}\;\text{scale}\right)}{\text{total}\;\text{number}\;\text{of}\;\text{calli}\times \text{the}\;\text{highest}\;\text{DSR}\;\text{scale}}\times 100. \end{array}$$

### 2.4. Tissue Sampling for Extractions

Samples were designated as follows: (i) callus inoculated with LXS081501 48 h prior to LXS080601 inoculation; (ii) callus inoculated with LXS081501; (iii) callus inoculated with LXS080601; (iv) callus inoculated with PDA as control. For each treatment, calli were sampled randomly at different time points (0, 24, 48, 72, 96, 120, and 144 h). Each treatment contained three replicates with each replicate having five calli. The whole experiment was repeated twice. Calli were arranged in sealed Petri dishes at 95% relative humidity and 25°C for 144 h. At the established time intervals, tissue cylinders (6 mm) from each callus were excised from the inoculation sites. In the case of inoculation with both LXS081501 and LXS080601, samples were only taken from LXS080601-inoculated sites. The excised tissues were rapidly frozen in liquid nitrogen and stored at −86°C until use for osmotic adjustment substance contents analysis and enzyme activity assays.

### 2.5. Determination of Osmotic Adjustment Substance Contents

Free proline was extracted according to Abrahám et al. [22] with some modifications. 1 g fresh weight of callus samples was homogenized in 5 mL 3% sulphosalicylic acid (w/v) on ice and the homogenates were centrifuged at 6000 g for 20 min at 4°C. 2 mL supernatant was mixed for reaction with 2 mL glacial acetic acid and 4 mL acid ninhydrin in the tube. After incubation at 100°C for 30 min, the reaction was terminated on ice. To the reaction mixture 5 mL toluene was added, which was then vortexed for 20 s and left on the bench for 5 min to allow the separation of the organic and water phases. The upper toluene phase was saved in a new tube for detection at the absorbance of 520 nm with toluene as blank reference. The proline concentration was determined by the standard curve and calculated as fresh weight (*μ*g/g FW).

The method to determine soluble sugar was modified from a procedure by Brugnoli [23]. 1 g callus samples were incubated in 2.5 mL distilled water at 100°C for 30 min. The samples were cooled on ice and then centrifuged at 10 000 g for 10 min at 4°C. The supernatant was saved and the pellet was resuspended in 2.5 mL water. After centrifugation, the two supernatants were combined and used for soluble sugar determination. 1 mL supernatant was transferred to a test tube, to which 1 mL 5% phenol was added and mixed thoroughly. 5 mL analytical grade sulphuric acid was then added to the tube with vertical agitation using a glass rod. The exothermic reaction was cooled in the air. Absorbance was recorded at 485 nm on spectrophotometer and the corresponding concentration was determined against a standard curve prepared by using a glucose solution (Sangon Co., Ltd., Shanghai, China). The amount of soluble sugar was expressed as *μ*g/g FW.

The determination of soluble protein was carried out with 1 g callus sample, which was homogenized in 5 mL extraction buffer (62.5 mM Tris-HCl, 0.5% SDS (w/v), 10% glycerin (w/v), and 0.5% *β*-mercaptoethanol (w/v), pH 7.6). The mixture was kept in agitation on ice for 30 min and then centrifuged at 10000 g for 10 min at 4°C with the supernatant being saved. The quantification of the soluble protein was determined at 595 nm with Coomassie Brilliant Blue G-250, and bovine serum albumin (Sangon Co., Ltd., Shanghai, China) was used to prepare standard curve [24].

### 2.6. Determination of Defense-Related Enzyme Activities

Phenylalanine ammonia lyase (PAL), peroxidase (POD), polyphenol oxidase (PPO), and catalase (CAT) were extracted according to the method of Moerschbacher et al. [25] with some modifications. Tissue samples (1 g) of each treatment were homogenized on ice in 2 mL of 100 mM sodium phosphate buffer (pH 5.8–8.8) containing 1% PVP (w/v). The homogenate was centrifuged at 12000 g for 20 min at 4°C, and the supernatant was used for the enzyme activity assays.

PAL activity was analyzed with modified method of Youssef et al. [26]. The reaction mixture, consisting of 0.5 mL crude enzyme extract and 2.5 mL L-phenylalanine (5 mM, in 100 mM sodium phosphate buffer, pH 8.8), was incubated for 60 min at 37°C. The reaction was ended by adding 1 mL of 6 N HCl. The amount of cinnamic acid produced was taken as a measure of enzyme activity using an increase in the absorbance of 0.01 at 290 nm. POD activity was determined using guaiacol as substrate [27]. The reaction mixture containing 0.5 mL supernatant preparation and 1.5 mL guaiacol (10 mM, in 100 mM sodium phosphate buffer, pH 6.8) was incubated for 30 min at room temperature. The increase in the absorbance at 470 nm was measured after 1 mL H_2_O_2_ (24 mM) was added.

PPO activity was determined by adding 0.5 mL enzyme extract to 2.5 mL catechol substrate (50 mM, in 100 mM sodium phosphate buffer, pH 5.8). The increase in the absorbance at 405 nm was measured [28]. CAT activity was reflected by the reduction in the amount of H_2_O_2_ according to the method of Cakmak and Marschner [29]. The reaction mixture (3 mL) contained 0.5 mL enzyme extract, 100 mM sodium phosphate buffer (pH 7.0), and 10 mM H_2_O_2_. The change in the absorbance at 240 nm was assayed. The activity of PAL, POD, PPO, and CAT was expressed as units per milligram of total protein per minute or hour (U/mg protein/min or U/mg protein/h), where one unit was defined as the change in absorbance per minute or per hour.

For *β*-1,3-glucanase and chitinase activity assay, 1 g tissue sample was homogenized on ice in 3 mL of 50 mM sodium acetate buffer (pH 5.0). The homogenate was centrifuged at 12000 g for 20 min at 4°C, and the supernatant was used for the enzyme activity assays.

*β*-1,3-Glucanase activity was determined using the method described by Wang et al. [30] with modifications. The enzyme activity was assayed by measuring the rate of reducing sugar production with laminarin (Sigma-Aldrich, Shanghai, China) as the substrate. The assay mixture consisted of 0.5 mL enzyme extract and 0.5 mL laminarin (1 mg/mL, dissolved with 50 mM sodium acetate buffer, pH 5.2). After 30 min incubation at 37°C, 2 mL of 3,5-dinitrosalicilate was added and the mixture was boiled at 100°C for 15 min. After cooling, the reducing sugars were measured spectrophotometrically at 492 nm. Glucose standards and enzyme or substrate blanks were included. Chitinase activity was measured with colloidal chitin as substrate [31]. Final activity values were expressed as units per milligram of total protein per minute or hour, where one unit was defined as the enzyme activity catalyzing the formation of 1 mmol glucose per minute or 1 *μ*mol of N-acetyl-glucosamine per hour for *β*-1,3-glucanase (U/mg protein/min) and chitinase (U/mg protein/h), respectively.

### 2.7. Data Analysis

All statistical analysis was carried out with SPSS software (Version 16.0, SPSS Inc., Shanghai, China). Analysis of variance (ANOVA) was performed to determine the statistical significance by Duncan's multiple range tests (*P* < 0.05).

## 3. Results

### 3.1. Induced Resistance against LXS08061 Infection by LXS081501 Treatment

**Figure 1:**
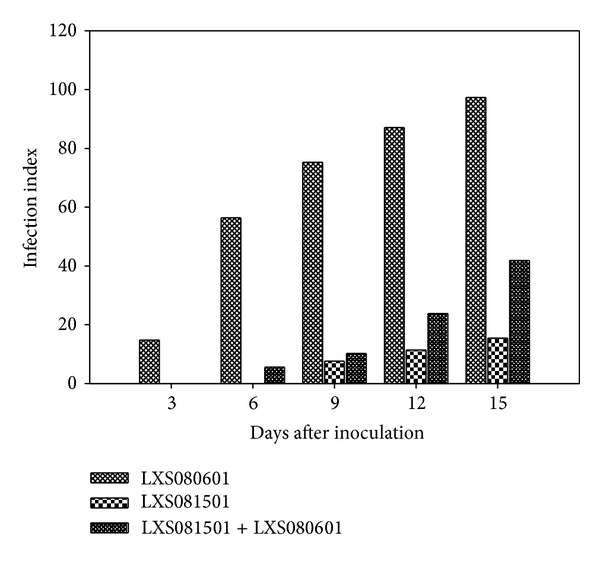
Induction of resistance in apple calli against the virulent* Vmm* strain LXS080601 by preinoculation with the attenuated* Vmm *strain LXS081501. Each column represents the average values of “Fuji” and “Gala” cultivars. Experiments included 30 calli per treatment and were repeated twice with three replicates.

To evaluate the induced resistance of the attenuated* Vmm* strain LXS081501, “Fuji” and “Gala” calli were pretreated with LXS081501 and then the virulent* Vmm* strain LXS080601 was inoculated. The result showed that pretreatment of LXS081501 significantly affected infection and symptom development by LXS080601 on apple calli (Figure 1). When the calli were inoculated with LXS080601 alone, the growth of hyphae and slight browning around the inoculation site were observed at 3 dai (infection index = 14.76), and then the hyphae expanded rapidly and browning degree was aggravated gradually. At 15 dpi, almost all the calli inoculated with LXS080601 alone were covered by dense hyphae and became severely water-soaked as well as dark-brown with the infection index up to 97.32. In contrast, pretreatment of calli with LXS081501 effectively mitigated the disease development caused by LXS080601 since no obvious symptoms were produced until 6 dpi (infection index = 5.62) and even at 15 dpi the infection index was only 41.81, much lower than when inoculated with LXS080601 alone. Calli treated with LXS081501 alone exhibited slight browning and even no noticeable hyphae growth was observed at 15 dpi (infection index = 15.42).

### 3.2. Temporal Dynamics of Osmotic Adjustment Substance Contents

Using calli as experimental plant material, the temporal profiles of free proline, soluble sugar, and soluble protein were analyzed in three treatments including inoculation with attenuated strain LXS081501 alone, with virulent strain LXS080601 alone, or with both. PDA inoculation served as control.

#### 3.2.1. Free Proline Analysis

**Figure 2:**
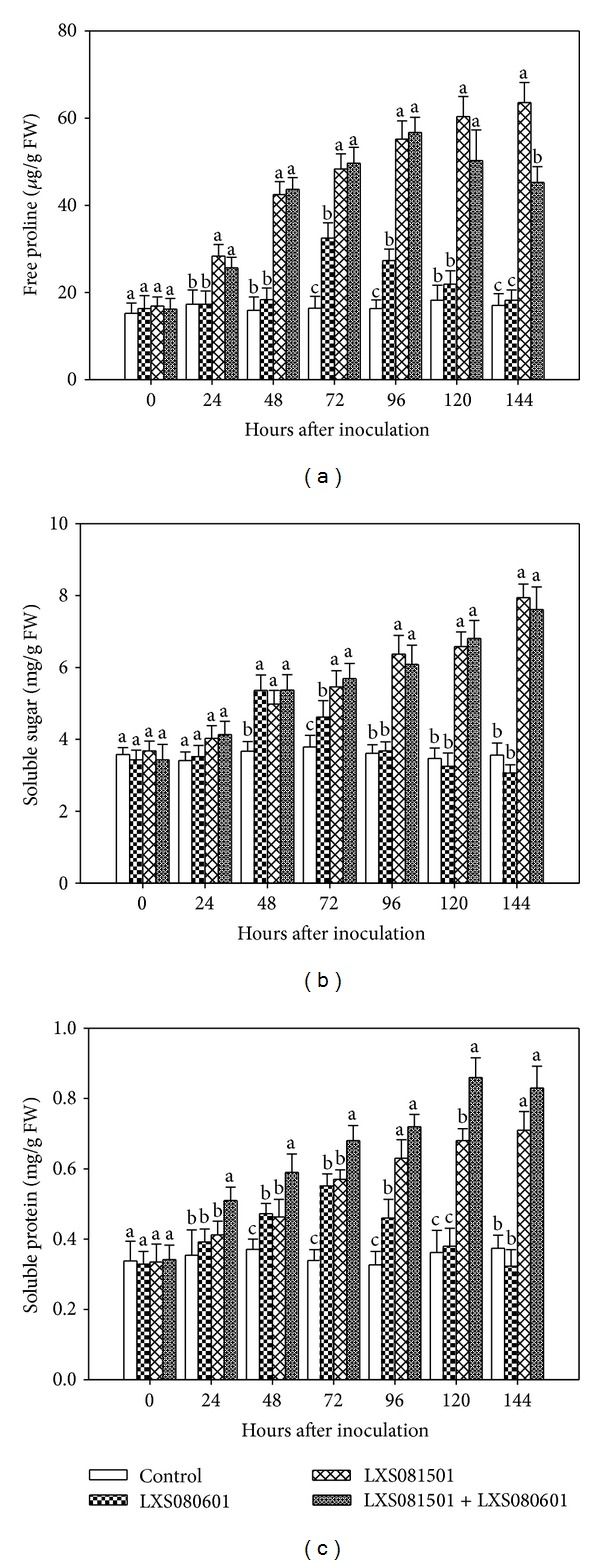
Changes in free proline content (*μ*g/g FW), soluble sugar, and soluble protein contents (mg/g FW) in the apple calli inoculated with PDA (control), LXS080601, LXS081501, and LXS081501 + LXS080601. Data represent the mean of “Fuji” and “Gala” cultivars in two independent experiments. Error bars represent standard error of the mean. Different letters on the bar show statistical significance (*P* < 0.05) in the osmotic adjustment substance contents at the indicated time point.

The levels of free proline were determined for each treatment. When calli were inoculated with attenuated strain LXS081501, free proline increased rapidly from 24 hai and maintained high levels at 144 hpi (63.58 *μ*g/g FW). Similar trends were observed for the free proline levels in calli pretreated with LXS081501 followed by LXS080601 infection. However, in the latter case, free proline slightly decreased after 96 hpi but still remained at high levels at 144 hpi (45.27 *μ*g/g FW). During early time points until 72 hours, the free proline of LXS080601 treated calli increased slowly, which declined to control level at 120 hpi. Overall, LXS080601-inoculated calli produced much less free proline compared with the other treatments. The control exhibited no significant change overtime (Figure 2(a)).

#### 3.2.2. Soluble Sugar Analysis

It was observed that, until 48 hpi, soluble sugar contents remained similar in three treatments, with slight increase compared with the control inoculation (Figure 2(b)). The soluble sugar content in calli inoculated with LXS080601 then declined quickly to the level of control at 96 hpi whereas those in LXS081501 or LXS081501 plus LXS080601 treated calli kept rising up to 144 hpi, with the levels 2.23 and 2.14 times higher than that in control, respectively.

#### 3.2.3. Soluble Protein Analysis

LXS080601-inoculated calli exhibited increased levels of soluble protein until 72 hpi (0.55 mg/g FW), and then the soluble protein content decreased to control level at 120 hpi. Calli inoculated with either LXS081501 or LXS081501 plus LXS080601 experienced continuous rise in soluble protein content, with the later treatment increasing more dramatically, reaching 0.86 mg/g FW at 120 hpi. It was noticeable that, until 72 hpi, the soluble protein levels were similar for LXS081501 and LXS080601 treatment; however, after that time point, soluble protein content for calli inoculated with LXS081501 kept accumulating while the other diminished to control level.

### 3.3. Temporal Dynamics of Enzyme Activities

#### 3.3.1. PAL, POD, PPO, and CAT Activities

**Figure 3:**
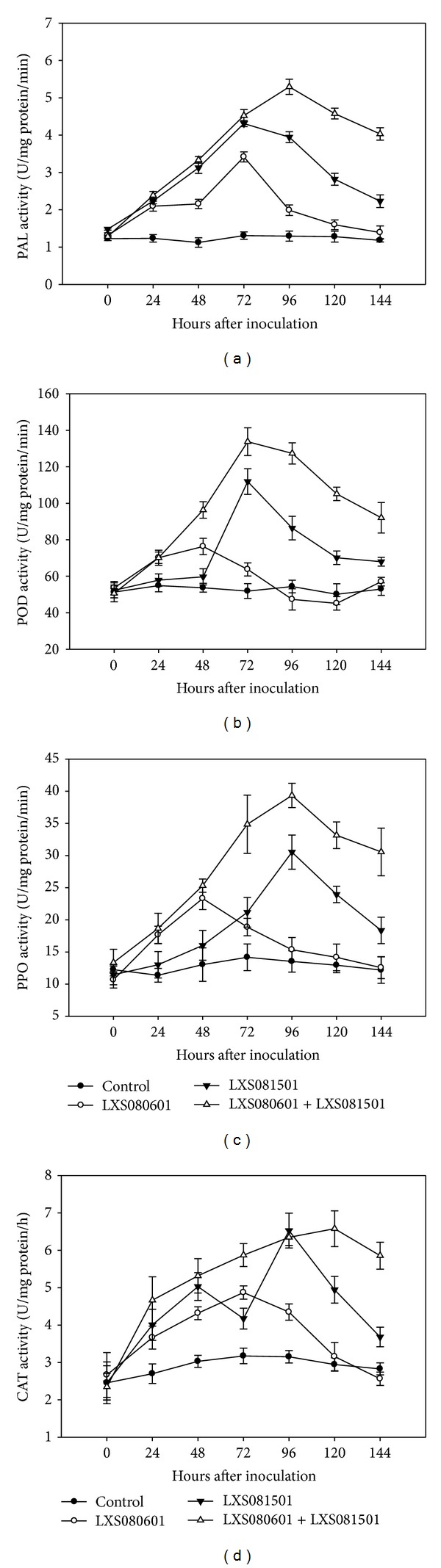
Time course of PAL (a), POD (b), PPO (c), and CAT (d) activities in extracts from apple calli inoculated with PDA (control), LXS080601, LXS081501, and LXS081501 + LXS080601. Data represent the mean of “Fuji” and “Gala” cultivars in two independent experiments. Error bars represent standard error of the mean.

Four enzymes, including PAL, POD, PPO and CAT, were selected to determine their activities for different treatments. All these enzymes remained unchanged activities for PDA inoculated calli, which served as control. The other three treatments (Figure 3) showed increased enzyme activities to varying degrees.

For PAL, its activity started to increase at an early time point (24 hpi) for all pathogen treated calli. Specifically, LXS080601 treated calli showed enhanced PAL activates until 72 hpi (2.61 times higher than control), since when the enzyme activity declined dramatically to control level at 144 hpi (1.39 U/mg protein/min). In contrast, when the calli were inoculated with LXS081501, PAL activity increased remarkably and peaked at 72 hpi (3.29 times higher than control). PAL then showed decreased activity but still remained highly active at 144 hpi compared with control or LXS080601 treatment. Substantial rise in PAL activity was observed for LXS081501 plus LXS080601 treatment (5.29 U/mg protein/min at 96 hpi).

POD activity for three treatments showed similar changing patterns, with activity peaking at different time points with varying levels (Figure 3(b)). In detail, LXS080601 treatment experienced increased POD activity starting from 24 hpi to 48 hpi, and then decreased activity was observed that became similar to control at 96 hpi (47.33 U/mg protein/min). Although having similar POD activity to LXS080601 treatment at 24 hpi, calli inoculated with LXS081501 plus LXS080601 exhibited exponential rise in POD activities until 72 hpi (133.75 U/mg protein/min), after when POD activity also declined measurably (92.04 U/mg protein/min at 144 hpi). Interestingly, POD activity for LXS081501 treatment remained stable and much lower than the other two treatments at early time points (0 hpi to 48 hpi); however, the activity then increased markedly, reaching maximum at 72 hpi (111.89 U/mg protein/min).

The changing patterns for PPO activity were similar to those of POD activity. As shown in Figure 3(c), LXS080601 and LXS081501 plus LXS080601 treated calli displayed comparable PPO activity from 0 hpi to 48 hpi, after which obvious difference appeared since the former experienced continuous declined PPO activity (15.35 U/mg protein/min at 96 hpi), while PPO activity for the latter kept increasing and peaked at 96 hpi (39.35 U/mg protein/min). An increase in PPO activity was also observed overtime in calli inoculated with LXS081501, with a peak at 96 hpi (30.53 U/mg protein/min).

Figure 3(d) depicted CAT activity in different treatments. It was observed that all the treatments had increased CAT activity starting from 24 hpi. LXS080601 treatment induced an increase in CAT activity reaching the maximum level at 72 hpi (1.54 times higher than control), since when CAT activity declined sharply to control level at 120 hpi (3.16 U/mg protein/h). However, LXS081501 plus LXS080601 treatment could sustain the increased level of CAT activity up to 120 hpi (2.24 times higher than control). In contrast to the other two treatments, CAT activity for LXS081501 treatment increased and peaked at 48 hpi (1.66 times higher than control), which was followed by another and higher peak at 96 hpi (2.07 times higher than control).

#### 3.3.2. *β*-1,3-Glucanase and Chitinase Activities

**Figure 4:**
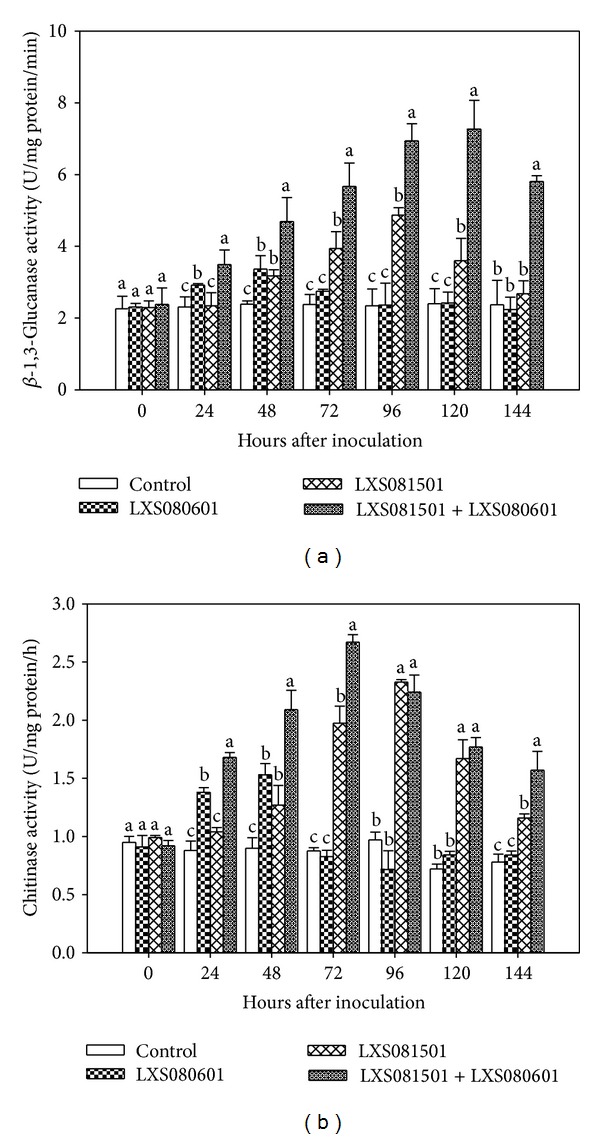
Time course of *β*-1,3-glucanase (a) and chitinase (b) activities in extracts from apple calli inoculated with PDA (control), LXS080601, LXS081501, and LXS081501+LXS080601. Data represent the mean of “Fuji” and “Gala” cultivars in two independent experiments. Error bars represent standard error of the mean. Different letters on the bar show statistical significance (*P* < 0.05) in *β*-1,3-glucanase and chitinase activities at the same hour.

For *β*-1,3-glucanase and chitinase activities, all treatments except control displayed various changing patterns. As shown in Figure 4, LXS080601 inoculated calli exhibited minor increase in both *β*-1,3-glucanase and chitinase activities at 24 and 48 hpi, and for the other following time points their activities were similar to those of control treatment. After LXS081501 inoculation, *β*-1,3-glucanase and chitinase activities increased significantly from 48 hpi and peaked at 96 hpi which was 2.08 and 2.40 times higher than control, respectively (Figure 4). Compared with the other two treatments, calli inoculated with LXS081501 plus LXS080601 displayed faster increase and thus higher *β*-1,3-glucanase and chitinase activities, which increased sharply from 24 hpi and peaked at 120 hpi for *β*-1,3-glucanase (3.03 times higher than control) and at 72 hpi for chitinase (3.05 times higher than control).

## 4. Discussion

*Valsa* canker disease is a typical apple trunk disease and the research is hindered due to the experimental variations caused by the branch ages, water content, positions on the tree, and the sampling times. Wei et al. [32] developed a simple and stable method for disease evaluation using detached apple leaves; however, the expanded apple leaves could only be obtained in fruit tree growing season. To circumvent these disadvantages, the present study successfully established a* Vmm*-apple callus interaction system to investigate* Valsa* canker disease. With culturing conditions amendable to manipulation, calli have good uniformity in growing status and can be propagated at any time, which minimizes variations and provides enough experimental materials. In order to evaluate the stability and reliability of* Vmm*-apple callus interaction system, we examined the pathogenicity of 28* Vmm* strains using “Fuji” calli, detached branches, and younger trees as experimental materials. Interestingly, the results were consistent in the three independent experiments. In addition, this interaction system allows for evaluating the resistant apple germplasm resources such as* M. hupehensis* Rehd.,* M. baccata* Borkh., and* M. sieboldii* Rehd., and the results were in line with the identification in field. Furthermore, the calli can be used to estimate the effects of cell wall degradation enzymes and toxic compounds produced by* Vmm *[7, 8], which provides an efficient way to study* Vmm *pathogenesis.

Previous researches revealed that resistance to pathogen infection can be induced in plants by avirulent or attenuated pathogens before treatment; however, such induced resistance rarely leads to complete disease control, resulting instead in a reduction in lesion size and/or number [13, 33–35]. Research in this area is thriving with the accumulation of many meaningful results. Recently, our group isolated an attenuated* Vmm* strain LXS081501 [8], the pretreatment of which was found to be effective in reducing the lesion size on apple branches caused by the virulent* Vmm* strain LXS080601 infection (see Supplement Table 1 available online at http://dx.doi.org/10.1155/2014/201382). In this study, when “Fuji” and “Gala” calli were pretreated with LXS081501, the disease symptoms and infection index produced by LXS080601 decreased significantly (Figure 1). Our result agreed with the report by Salazar et al. [14] with strawberry, suggesting that induced resistance may be responsible for the observed disease reduction. To understand this resistance mechanism, osmotic adjustment substances and enzymes related to plant defense were evaluated in this current study.

It is known that plant cell metabolism is manipulated by pathogen invasion, and, to survive these unfavorable conditions, plant cells accumulate higher level of osmotic adjustment substances [36–38]. Proline, soluble sugars, and soluble proteins are among those osmotic adjustment substances that are involved in subcellular structures and protein protection, energy supply, and antioxidation [36, 39, 40]. Song and Zheng [41] reported that the induced resistance in cotton seedlings against* Fusarium* wilt was related to the accumulation of proline in tissues. In addition, elicitors or pathogens could elevate the contents of proline, soluble sugars, and proteins in plants [42, 43]. Our results indicated that all these osmolytes could be induced by* Vmm* infection; however, their increasing levels were significantly different between three treatments with the induction effect of LXS081501 better than that of LXS080601 (Figure 2).

Extensive studies have shown that avirulent pathogen could elicit the expression of a range of genes, especially those encoding defense-related enzymes including PAL, PPO, POD, and CAT and those encoding pathogenesis-related proteins including chitinase and *β*-1,3-glucanase, leading to induced resistance in plants [13, 14, 16, 44]. In the present study, the analysis of the accumulation kinetics of the selected enzymes in treated apple calli showed that* Vmm* infection induced an increase in the activity of all tested enzymes compared with controls. Among these analyzed enzymes, PAL showed the greatest induction in response to* Vmm* inoculation, especially to LXS081501 in combination with LXS080601 treatment (Figure 3(a)). Interestingly, PAL is considered important in host defense, since it is involved in several metabolic pathways including the phenylpropanoid pathway, from which scoparone and scopoletin are derived [26]. Concerning PPO, a peak of activity was detected in LXS081501 or LXS081501 plus LXS080601-inoculated calli at 96 hpi and in LXS080601-inoculated calli at 48 hpi, which was in agreement with other findings that PPO activities could be induced by elicitors or pathogen infection [28, 45]. Furthermore, PPO catalyzes the oxygen-dependent oxidation of phenols to quinones and enhanced pathogen resistance was acquired in the transgenic tomato plants overexpressing PPO [46].

POD and CAT are two important hydrogen peroxide scavengers* in planta *that could reduce the toxic effects of reactive oxygen species (ROS) on plant cells [25, 36]. He [44] reported that avirulent* Rhizoctonia* treatment increased POD and PAL activities and the elevated levels were positively correlated with the disease resistance in rice. In this study, LXS081501 in combination with LXS080601 also markedly induced POD and CAT activities (Figure 3). With respect to the other two enzymes, the increases in chitinase and *β*-1,3-glucanase activities were particularly interesting (Figure 4). As important pathogenesis-related (PR) proteins, chitinase and *β*-1,3-glucanase are able to degrade fungal cell wall composed of chitin and *β*-glucan, which exhibit antifungal properties including delaying growth of hyphae and spore germination as well as decreasing the incidence of pathogen infection [47]. Similarly, elicitor or pathogen also could induce a significant increase in chitinase and *β*-1,3-glucanase activities in plants [13, 26, 27].

Finally, when we took into account all the results obtained in this study, we found LXS081501 or LXS081501 plus LXS080601 treatment to be more effective at inducing the contents of osmotic adjustment substances and the activities of defense-related enzymes than LXS080601 inoculation. Moreover, in calli inoculated with LXS081501 in combination with LXS080601, the contents of osmotic adjustment substances and the activities of enzymes reached the peaks at different times, and they always maintained much higher levels than control until 144 hpi. Although the osmotic adjustment substance contents and enzyme activities in apple calli inoculated with LXS080601 alone reached the maximum at 48 or 72 hpi, they declined dramatically to control levels before 144 hpi. These results might explain the phenotypes shown in Figure 1 where apple calli inoculated with LXS080601 alone only exhibited slight hyphae growth and browning at 3 dpi, and then the hyphae expanded rapidly with browning becoming more severe (infection index = 56.38 at 6 dpi). In contrast, the calli pretreated with LXS0801501 48 h prior to the inoculation of LXS080601 showed no obvious symptoms at 3 dpi, and only a few of the calli produced slight browning in proximity to the inoculation site at 6 dpi (infection index = 5.62). Thus, we conclude that pretreatment of LXS0801501 could elicit stronger reactions in calli and thus is more effective in limiting LXS080601 colonization and development.

## 5. Conclusion

In the present study, a* Vmm*-apple callus interaction system was developed to investigate* Valsa* canker disease and the pathogen. Utilizing this system, we examined the induced resistance of the attenuated* Vmm* strain LXS081501 in apple calli against further infection by the virulent* Vmm* strain LXS080601. A detailed characterization of several important aspects involved in induced resistance of LXS081501 was carried out. The accumulation of osmotic adjustment substances and the activation of several highly coordinated defense-related enzymes might be able to inhibit, directly or indirectly, the pathogen infection and development. This finding represents the first report of an induced resistance by attenuated* Vmm* strain in apple and allows us to envision the practical potentials of using* Vmm *strain LXS081501 to control* Valsa* canker disease in apple or other fruit trees.

## Supplementary Material

To evaluate the induced resistance of the attenuated *Vmm* strain LXS081501, ‘Fuji' and ‘Gala' detached branches and younger trees were pretreated with LXS081501 and then the virulent *Vmm* strain LXS080601 was inoculated. The result showed that pretreatment of LXS081501 significantly affected the lesion size caused by LXS080601 both on ‘Fuji' and ‘Gala' branches (Table 1). When ‘Fuji' and ‘Gala' younger trees were inoculated with LXS080601 alone, the lesion sizes were 5.31 cm^2^ and 3.93 cm^2^ at 15 dpi, respectively. In contrast, pretreatment of ‘Fuji' and ‘Gala' younger trees with LXS081501 effectively mitigated the disease development caused by LXS080601 since the lesion sizes were only 1.11 cm^2^ and 1.15 cm^2^, respectively, much smaller than when inoculated with LXS080601 alone.
