# Supplementary material for: Induction of Resistance Mediated by an Attenuated Strain of Valsa mali var. mali Using Pathogen-Apple Callus Interaction System
Source: ScientificWorldJournal. 2014 Jun 25;2014:201382. doi: 10.1155/2014/201382 (PMC4098620; doi:10.1155/2014/201382)
Supplement: Supplementary file 1 — To evaluate the induced resistance of the attenuated Vmm strain LXS081501, ‘Fuji' and ‘Gala' detached branches and younger trees were pretreated with LXS081501 and then the virulent Vmm strain LXS080601 was inoculated. The result showed that pretreatment of LXS081501 significantly affected the lesion size caused by LXS080601 both on ‘Fuji' and ‘Gala' branches (Table 1). When ‘Fuji' and ‘Gala' younger trees were inoculated with LXS080601 alone, the lesion sizes were 5.31 cm2 and 3.93 cm2 at 15 dpi, respectively. In contrast, pretreatment of ‘Fuji' and ‘Gala' younger trees with LXS081501 effectively mitigated the disease development caused by LXS080601 since the lesion sizes were only 1.11 cm2 and 1.15 cm2, respectively, much smaller than when inoculated with LXS080601 alone. [file 201382.f1.docx]

**Supplementary Table**

TABLE 1: Lesion sizes produced by *Vmm* strains LXS081501, LXS080601 and LXS081501+LXS080601 in ‘Fuji’ and ‘Gala’ detached branches (2 years old) and younger trees (5 years old).

| *Vmm* Strains | Lesion size on ‘Fuji’ (cm^2^) | |  | Lesion size on ‘Gala’ (cm^2^) | |
| --- | --- | --- | --- | --- | --- |
|  | detached branch | younger tree |  | detached branch | younger tree |
| LXS081501 | 1.48±0.52 a | 0.95±0.40 a |  | 1.27±0.38 a | 0.86±0.45 a |
| LXS080601 | 9.67±1.26 b | 5.31±1.79 b |  | 7.06±1.95 b | 3.93±1.58 b |
| LXS081501+LXS080601 | 1.88±0.48 a | 1.11±0.63 a |  | 1.66±0.57 a | 1.15±0.61 a |

The lesion sizes were determined 15 days post inoculation. Different letters in the same column indicate significant differences (*P*<0.05) by Duncan’s multiple range test.
